# Changes in emergence phenology, fatty acid composition, and xenobiotic‐metabolizing enzyme expression is associated with increased insecticide resistance in the Colorado potato beetle

**DOI:** 10.1002/arch.21630

**Published:** 2019-10-16

**Authors:** Justin Clements, Jake M. Olson, Benjamin Sanchez‐Sedillo, Benjamin Bradford, Russell L. Groves

**Affiliations:** ^1^ Department of Entomology University of Wisconsin‐Madison Madison Wisconsin; ^2^ Department of Animal Sciences University of Wisconsin‐Madison Madison Wisconsin

**Keywords:** behavioral resistance, Colorado potato beetle, fatty acids, insecticide resistance

## Abstract

The Colorado potato beetle (*Leptinotarsa decemlineata*) is a major agricultural pest of solanaceous crops. An effective management strategy employed by agricultural producers to control this pest species is the use of systemic insecticides. Recent emphasis has been placed on the use of neonicotinoid insecticides. Despite efforts to curb resistance development through integrated pest management approaches, resistance to neonicotinoids in *L. decemlineata* populations continues to increase. One contributing factor may be alterations in insect fatty acids, which have multiple metabolic functions and are associated with the synthesis of xenobiotic‐metabolizing enzymes to mitigate the effects of insecticide exposure. In this study, we analyzed the fatty acid composition of *L. decemlineata* populations collected from an organic production field and from a commercially managed field to determine if fatty acid composition varied between the two populations. We demonstrate that a population of *L. decemlineata* that has a history of systemic neonicotinoid exposure (commercially managed) has a different lipid composition and differential expression of known metabolic detoxification mechanisms relative to a population that has not been exposed to neonicotinoids (organically managed). The fatty acid data indicated an upregulation of Δ^6^ desaturase in the commercially managed *L. decemlineata* population and suggest a role for eicosanoids and associated metabolic enzymes as potential modulators of insecticide resistance. We further observed a pattern of delayed emergence within the commercially managed population compared with the organically managed population. Variations in emergence timing together with specific fatty acid regulation may significantly influence the capacity of *L. decemlineata* to develop insecticide resistance.

## Research Highlights

- Organic and conventional management practices influence lipid compositions in the specialist herbivore Leptinotarsa decemlineata.
- Individuals resistant to imidacloprid overexpress known detoxification pathways relative to susceptible individuals.

## Graphical abstract

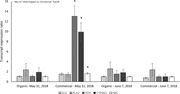

A commercially managed field population of Leptinotarsa decemlineata significantly overexpresses transcripts related to xenobiotic metabolism, including a cytochrome p450 6k1, ATP‐binding cassette subfamily G, and peroxidase‐like enzyme, when compared to an organically managed field population.

## 1 INTRODUCTION

The Colorado potato beetle, *Leptinotarsa decemlineata* Say (Coleoptera: Chrysomelidae), is a pest of multiple Solanaceous crops including potatoes, tomatoes, and eggplants. *L. decemlineata* populations are infamous for their ability to develop insecticide resistance to all major classes of insecticidal compounds labeled for their control (Alyokhin, Baker, Mota‐Sanchez, Dively, & Grafius, 2008; Whalon, Mota‐Sanchez, & Hollingworth, 2019). A common method used to control these insect pests is the utilization of at‐planting applications of systemic neonicotinoid insecticides classified in the mode of action Group 4A (IRAC International MoA Working Group, 2018). When first registered in 1995 and applied at planting to potato seed, the neonicotinoid group of insecticides provided growers with nearly season‐long control of beetle populations (Huseth & Groves, 2013). Since its initial registration, multiple beetle populations have developed significant levels of resistance to compounds in the neonicotinoid group (Alyokhin et al., 2006; Clements, Schoville, Peterson, Lan, & Groves, 2016b; Szendrei, Grafius, Byrne, & Ziegler, 2012; Zhao, Bishop, & Grafius, 2000). Multiple studies have explored the biological mechanisms that *L. decemlineata* use to overcome current insecticide treatments (Clements et al., 2016b; Clements, Sanchez‐Sedillo, Bradfield, & Groves, 2018; Clements, Schoville, Clements, Chapman, & Groves, 2016a; Crossley, Chen, Groves, & Schoville, 2017; Kaplanoglu, Chapman, Scott, & Donly, 2017; Mota‐Sanchez, Hollingworth, Grafius, & Moyer, 2006; Schoville et al., 2018; Zhu, Moural, Nelson, & Palli, 2016). Most of these studies focus on understanding the insect's ability to upregulate certain enzymatic detoxification mechanisms which are key to surviving exposure to neonicotinoid insecticides. Although the underlying physiological mechanisms for resistance have been examined, producers have also observed variation in the temporal patterns of *L. decemlineata* emergence among populations. Because the concentrations of neonicotinoid insecticides in potato decline over time after at‐plant applications (Huseth, Lindholm, Groves, & Groves, 2014), we hypothesize that this decline in concentration over time could favor variation in *L. decemlineata* emergence patterns resulting in delayed emergence to avoid lethal concentrations of neonicotinoid insecticides present in plants shortly after at‐plant applications.

In *L. decemlineata*, the role of physiological factors conferring fitness advantages for resistance have been examined. However, the clear ecological connections between biological factors that favor resistant phenotypes in potato agroecosystems are less understood, suggesting a need for further investigation of factors that may increase the fitness of resistant *L. decemlineata*. Fatty acid biosynthesis, metabolism, and catabolism in Coleopterans are involved in homeostatic (Machado et al., 2007), reproductive (Bowman, 2008), and developmental (Kort, 1990; Yocum, Buckner, & Fatland, 2011) processes that directly contribute to fitness. Most recently, Yocum et al. (2011) reported an increase in lipid biosynthesis in diapausing *L. decemlineata* compared to nondiapausing *L. decemlineata*, suggesting regulation of fatty acid biosynthesis as a requirement for diapause. Lehmann, Lyytinen, Sinisalo, and Lindström (2012) corroborated the findings of Yocum et al. (2011) and reported on the specific changes in the fatty acid composition of adult *L. decemlineata* over 10 days postemergence. Although the fatty acid composition was not a differentiating factor between diapausing and nondiapausing adults, it is not known if postdiapause fatty acid composition varies between insecticide‐resistant and susceptible individuals. Provided total lipids are maintained for survival throughout diapause, specific fatty acids, and fatty acid metabolic pathways may presumably be selectively utilized to aid in facilitating xenobiotic detoxification. Fatty acid metabolism contributes to antioxidant, antimicrobial, and detoxification pathways in *L. decemlineata*. Specifically, arachidonic acid (ARA, 20:4n‐6) is a substrate for cytochrome P450 (CYP) enzymes and regulates CYP expression (Panigrahy, Kaipainen, Greene, & Huang, 2010), which has a well‐documented role in insecticide detoxification pathways (Arnold et al., 2010; Kaplanoglu et al., 2017; Schoville et al., 2018; Scott, Liu, & Wen, 1998; Spector & Kim, 2015). Pan et al. (2015) demonstrated fatty acid biosynthesis pathways enriched in the presence of a thiamethoxam stressor in the cotton aphid, and further suggest the importance of fatty acid composition as a biological contributor to insecticide resistance. In this study, we explored this relationship in the Colorado potato beetle by analyzing the fatty acid compositions of beetles collected from both organic (not exposed to neonicotinoids) and commercial (exposed to neonicotinoids) potato fields over consecutive emergence windows to explore differences in fatty acid composition and its relationship to insecticide resistance.

To further build on this idea, we also hypothesize that *L. decemlineata* has concurrently evolved behavioral mechanisms to avoid insecticide exposure through changes in behavioral emergence patterns to minimize insecticide exposure in time. Behavioral resistance to insecticides is defined as the ability of an insect to avoid a lethal dose of insecticide (Yu, 2015). This adaptation can be through space, and as we suggest, through avoidance in time. Behavioral resistance has historically been associated with an insect‘s ability to become hypersensitive and detect insecticide concentrations (Yu, 2015). Sparks, Lockwood, Byford, Graves, and Leonard (1989) observed a dose‐dependent shift of horn flies (*Haematobia irritans*) away from pyrethroid‐treated ear tags to host bellies. Fray et al. observed a dietary preference in a green peach aphid (*Myzus pericae*) when presented the choice of thiamethoxam‐treated and untreated foliage where insects preferred to feed on clean foliage (Fray et al., 2014). Alyokhin and Ferro (1999) observed that insects with a prior history of insecticide resistance had the propensity to spatially avoid *Bacillus thuringiensis tenebrionis* (Btt)‐treated crops compared to untreated crops. Huseth and Groves (2013) hypothesized that a prolonged adult emergence of *L. decemlineata* from diapause was related to neonicotinoid resistance status and the driver of this effect was linked to chronic exposure to nonlethal doses of insecticide. In northern corn rootworm (*Diabrotica barberi*) extended diapause has previously been described, which allows the insect to circumnavigate crop rotation (Monsanto Company, 2014). The same phenomenon has been observed in *L. decemlineata*, as Tauber and Tauber (2002) observed the ability of *L. decemlineata* to have prolonged dormancy which has a vast impact on the effectiveness of crop rotation. Previous studies have also documented that the temporal concentration of in‐furrow and seed‐applied neonicotinoid applications follow a predictable decline in insecticide concentration in plant tissue over a 15‐day period (Huseth et al., 2014). While prior behavioral studies involving *L. decemlineata* did not reveal significant relationships of emergence phenology to insecticide resistance history (Huseth & Groves, 2013), it is plausible that the phenotypic variation among individuals within these selected populations may have limited the power to detect significant population‐level variation in postdiapause emergence phenology as a result of resistance status. It is possible that if *L. decemlineata* individuals which emerge from diapause later in the season tend to survive sublethal insecticide levels in the plant, this effect could favor the coevolution of tandem resistance mechanisms that are linked to both behavioral (diapause duration) and physiological (insecticide detoxification) factors that favor fitness of these phenotypes over a period of two decades of continuous systemic neonicotinoid use. To more accurately document the phenotypic variation within selected populations, an examination of the underlying physiological mechanisms coupled with the emergence phenology represents a novel approach to understand the relationship of insect physiological and behavioral mechanisms of resistance.

In the current study, our goals were to document the temporal patterns of *L. decemlineata* emergence and characterize the fatty acid composition of individuals at different emergence time points. We hypothesize that populations of *L*. *decemlineata* with a documented history of neonicotinoid resistance may vary in emergence phenology compared to a population with no prior exposure to systemic insecticides. Fatty acid compositions were then determined from replicate sets of individuals obtained at different time intervals representing the emergence period of each population. Median lethal concentration assays were performed, fatty acid composition analyses were conducted, and transcript regulation of known detoxification mechanisms and fatty acids were determined at each time point of emergence for the field populations. Specifically, we investigated the genetic regulation of known detoxification mechanisms, including a unique cytochrome P450, an adenosine triphosphate (ATP)‐binding protein, and lipid regulatory pathways.

## 2 MATERIALS AND METHODS

### 2.1 Ethics statement

No specific permits were required for field collection or experimental treatment of *L. decemlineata* for the study described.

### 2.2 Insect collection

Two populations of *L. decemlineata* were selected for analysis based on their prior history of insecticide inputs. The first population was obtained from an organically‐certified agricultural operation in south‐central Wisconsin and had no history of systemic insecticide applications, specifically with no use of neonicotinoids. The second population was obtained from a commercially managed potato field in central Wisconsin with a 20‐year history of systemic neonicotinoid at‐plant applications. It has been previously estimated that *L. decemlineata* movement is annually limited to 400 m (Sexson & Wyman, 2005) and locations of fields chosen for the current study were separated by more than 400 m from other potatoes to avoid confounding effects due to immigration. Both fields were monitored daily for *L. decemlineata* emergence until first potato colonization was noted at each location. After the onset of initial colonization, insects were given 1 week to colonize the potato crop. After the first week of colonization, all adult insects were removed from a preselected section of each experimental field and individuals were placed in sterile plastic containers. For the purposes of this investigation, the timing of initial insect colonization date of the crop was used as a reference, as the true emergence data from the soil were not known. At the organically managed field location, all adult insects were collected from the entire field (0.13 ha). At the commercially managed field location, all adult insects were collected from a 0.16 ha edge section of the field. For five successive weeks following the onset of initial colonization, all adult beetles present in each field section were collected and brought back to the University of Wisconsin‐Madison for further analysis. Experimental field sections were located adjacent to field margins containing habitat conducive for overwintering adult *L. decemlineata*. Over successive weeks following collection, newly emerged adult insects were allowed to recolonize the same field sections. Collections were continued throughout the first generation of *L. decemlineata* (emerging overwintered adults) and degree‐day accumulations (base 52°F) were computed from nearby weather stations (commercial site: NKAW3 Rome station, elevation 1017 ft, lat/long: 44.2567°N, −89.8100°W; organic site: EW5990 Vermont Township station, elevation 1,110 ft, lat/long: 43.0831°N, 89.7831). Weather stations were located approximately 20 km from field locations.

Insects collected from each field location were processed within 8 hr of collections. Insects from each population were counted to determine insects emerged per hectare per week and used to calculate a cumulative emergence proportion. Twelve insects from each population were frozen in liquid nitrogen and stored at −80°C for either fatty acid or transcript abundance analysis per collection interval. Remaining adult insects were placed on nontreated potato foliage for 72 hr and used in imidacloprid median lethal concentration assays.

### 2.3 Imidacloprid median lethal concentration assay

Adult *L. decemlineata* beetles collected weekly at each field location were assayed for median lethal concentration (LC_50_) using technical imidacloprid (technical grade, 98.8%, Bayer Crop Sciences, Kansas City, MO). Beetles were bioassayed using a topically applied, 1 μl solution of imidacloprid carried in acetone ranging in concentrations between 0 and 50 µg/µl applied to the ventral surface of the abdomen between the first and second sternites. After topical application, adult beetles were placed in Petri dishes in an incubator held at 26°C, 70% humidity, and a 16:8 hr light and dark cycle, and given fresh untreated potato foliage daily day for 7 days. After 7 days, the number of live beetles incapacitated beetles, and dead beetles were recorded, as measured by the pencil test (Zhao et al., 2000). Briefly, adult beetles were presented with the opportunity to climb a pencil: if they could move a full body length they were considered alive, if they appeared alive but could not move a body length they were considered incapacitated, and if they had no movement, even after pinching their back legs with tweezers, they were considered dead. Incapacitated and dead beetles were pooled and LC_50_ values for each population were calculated. The median lethal concentration (LC_50_) of the adult beetles was estimated for each field location over the entire first generation. A probit regression analysis was conducted (PROC PROBIT, SAS), and was used to assess the phenotypic response to insecticide exposure. To assess the overall imidacloprid susceptibility for each field an LC_50_ statistical analysis was conducted, we calculated the ratio of dead insects to total insects for each insecticide concentration in the LC_50_ assay of each population. This ratio was used to create a representative population with an equal number of individuals to conduct a PROC PROBIT regression (PROC PROBIT, SAS).

### 2.4 Tissue fat extraction and fatty acid composition analysis

The whole‐body total fat of individual adult *L. decemlineata* (*n* = 5/group) was extracted according to the Folch methodology using dichloromethane as a substitute for chloroform (Folch, Lees, & Sloane Stanley, 1957). Total extracted fatty acids were methylated using 0.5 M sodium methoxide, similar to methods described by Christie (Christie, 1982) using select modifications described by Politz, Lennen, and Pfleger (2013). Briefly, toluene was added to dried dichloromethane extract (2:1 vol/wt). Next, 0.5 M sodium methoxide was added to lipid extracts (100:1 vol/wt) and samples were heated at 60°C for 10 min in a water bath. The methylation reaction was arrested with 0.35 M glacial acetic acid (1.5:1 volume/volume) followed by hexane extraction of fatty acid methyl esters (FAME) to yield a final FAME concentration of 10 mg/ml for composition analysis by gas chromatography. The relative abundance of FAMEs was analyzed using gas chromatography (Agilent 6890N) coupled with flame ionization detection as previously described (Huebner et al., 2010). A 100 m biscyanopropyl polysiloxane capillary column (Rt‐2560; Restek Corp, Bellefonte, PA) was used for separation of FAMEs. FAMEs were identified using a custom qualitative FAME standard (#SPL4833; Matreya LLC, Pleasant Gap, PA). The delta‐(Δ)^6^ and Δ^9^‐desaturation indices, rate indicators of specific fatty acid metabolism, were calculated from fatty acid relative abundance data (Sampath & Ntambi, 2006). Specifically, the Δ^6^‐desaturation index was calculated as ([20:4n‐6]/[20:4n‐6 + 18:2n‐6]), while the Δ^9^‐desaturation index was calculated as ([16:1c9 + 18:1c9]/[16:1c9 + 18:1c9 + 16:0 + 18:0]).

FAME relative abundance data were analyzed by one‐way analysis of variance and subsequently, Tukey's HSD to determine differences between treatments (SAS; SAS Institute, Cary, NC). When Levene's test for homogeneity of variances was significant, data were analyzed by the MIXED procedure with multiple mean square errors. Post hoc analysis for the MIXED procedure was performed using LSD. All statistical tests were considered significant at *p* < .05.

### 2.5 Differential transcript abundance analysis

Transcript abundance of five target genes previously shown to be important in imidacloprid‐resistance (ATP‐binding cassette subfamily G, cytochrome p450 6k1, peroxidase‐like enzyme) and fatty acid metabolism (elongation of very‐long‐chain fatty acid enzyme, phospholipase A2‐like enzyme) were examined to observe differences in transcript regulation between the insecticide susceptible and resistant populations among the different time points of collection. Total RNA was extracted from each adult with Trizol (Life Technology, Grand Island, NY). DNA contamination was removed with Turbo DNase (Life Technology) and total RNA was purified through EtOH precipitation, air‐dried until no visible liquid was observed, and then suspended in 50 µl DNase/RNase‐free H_2_O. All RNA concentrations were equalized before input into the cDNA synthesis kit, and the subsequent complementary DNA (cDNA) was generated with a SuperScript III Kit (Thermo Fisher Scientific). The cDNA was diluted to a final concentration of 5 ng/µl RNA equivalent input for quantitative PCR (qPCR). Ribosomal protein 4 was used as a reference gene in the analysis. The qPCR reaction was run on a CFX‐96 platform (Bio‐Rad Laboratories, Hercules, CA) with a master mix of Bullseye EverGreen (MIDSCI, Valley Park, MO). The qPCR reactions were conducted using the Pfaffl efficiency calibrated methodology; primer and primer efficiency (Pfaffl, 2001) can be found in Table S1. Triplicate reactions were run at 95°C for 10 min, followed by 95°C for 15 s, and 62°C for 60 s for a total of 40 cycles. The methodology described by Bradburn ([Link]) was used to determine transcript abundance. Ribosomal protein 4 was used as the baseline for messenger RNA transcript expression for transcripts of interest. Transcript expression was then statistically evaluated between populations using the organic population as the reference control. Mean C_*t*_ values were collected for each biological replicate and fold change estimates between the organic and commercial populations were calculated. A two‐tailed Student's *t* test was conducted between treatment groups with a *p* < .05 considered as statistically significant.

## 3 RESULTS

### 3.1 Imidacloprid median lethal concentration assay

Median lethal concentration assays (LC_50_) using imidacloprid demonstrated a difference in imidacloprid susceptibility between the organic and commercial potato field populations of *L. decemlineata*. Median lethal concentration assays were performed weekly on populations of adult *L. decemlineata* at both locations over the first generation emergence period. The organically managed population of *L. decemlineata*, which had no prior exposure to imidacloprid, resulted in LC_50_ estimates that ranged from 1.64 to 2.82 µg imidacloprid per beetle over the 5‐week sampling interval, while the commercially managed population of insects, with a history of neonicotinoid insecticide exposure, resulted in LC_50_ estimates ranging from 2.00 to 12.12 µg imidacloprid over the same sampling interval (Table 1). To access the overall imidacloprid susceptibility of both fields monitored, a consolidated LC_50_ estimate was generated across all collection dates resulting in an estimated LC_50_ of 2.32 µg at the organically managed field and 6.15 µg imidacloprid at the commercially managed field. Averaging over the 5‐week collection interval at both locations, the commercially managed population was statistically more resistant (3‐fold) to imidacloprid than the organically managed population.

**Table 1:** Median lethal concentration (LC_50_) estimates of weekly collections and cumulative totals for populations obtained from an organically and commercially managed potato field in Wisconsin, 2018

Population	Week	N	Slope	LC50 (μg)	95% CI^a^	*χ* ^2^ ^b^	PR > *χ* ^2^
Commercial	1 (May 31)	200	1.12	2.00	(0.58–4.14)	19.20	<0.001
Commercial	2 (Jun 7)	150	2.02	7.16	(0.49–12.14)	6.08	0.0137
Commercial	3 (Jun 14)	150	1.50	6.44	(3.60–11.05)	31.59	<0.0001
Commercial	4 (Jun 21)	100	1.86	12.12	(6.26–23.09)	19.52	<0.0001
Commercial cumulative	NA	NA	1.47	6.15	(4.83–7.69)	158.10	<0.001
Organic	1 (May 31)	350	1.10	1.64	(1.06–2.55)	79.80	<0.001
Organic	2 (Jun 7)	150	2.48	2.03	(1.27–3.16)	24.94	<0.001
Organic	3 (Jun 14)	70	2.28	2.82	(0.17–6.22)	5.63	0.017
Organic	4 (Jun 21)	0	NA	NA	NA	NA	NA
Organic cumulative	NA	NA	1.83	2.32	(1.81–2.87)	92.00	<0.001

^a^ 95% confidence interval (CI) estimates around LC_50_ value estimates.

^b^ Chi‐square analysis effects of the Proc Probit regression.

### 3.2 Fatty acid composition analysis

Fatty acid composition in adult *L. decemlineata* significantly changed within the commercially managed potato field location over time, while composition within adult beetles was not significantly different over time in the organically managed field location. The analysis of whole‐body fatty acids in the organic *L. decemlineata* population over consecutive emergence weeks shows few differences exist in composition over time (Table 2). There were no meaningful differences in the main fatty acid classes of saturated fatty acids, monounsaturated fatty acids (MUFA), and polyunsaturated fatty acids (PUFA) between early‐ compared to later‐emerging *L. decemlineata*. Furthermore, the Δ^9^ and Δ^6^‐desaturase indices were unchanged across beetle collection times at different weeks of emergence.

**Table 2:** Susceptible (organically managed) *L. decemlineata* population fatty acid composition over time

Week	1	2	3	4	*p* value
Fatty acid	Relative abundance (g/100 g FAME)				
14:0	1.3^b^	2.2^a^	2.4^a^	0.6^c^	<.01
16:0	11.2	11.6	9.3	12.4	.37
18:0	7.7	5.8	7.9	6.1	.33
20:0	1.3	1.3	1.4	1.2	.85
∑SFA	21.5	21.0	21.0	20.3	.53
16:1 c9	0.4	0.8	0.4	0.8	.09
18:1 c9	37.0	32.9	29.0	38.6	.24
18:1 c11	1.6	1.3	1.8	1.2	.39
∑MUFA	38.7	35.0	31.2	40.3	.27
18:2n‐6	17.3	21.6	22.4	18.2	.47
18:3n‐3	11.5	12.2	13.2	12.7	.64
20:4n‐6	0.6	1.1	1.0	0.9	.24
∑PUFA	32.0	35.9	37.5	33.0	.44
Unknown	9.9	8.4	10.4	6.5	.24
Δ^6^ index^3^	0.03	0.05	0.04	0.05	.20
Δ^9^ index^4^	0.66	0.66	0.63	0.67	.61

Abbreviations: FAME, fatty acid methyl esters; MUFA, monounsaturated fatty acids; PUFA, polyunsaturated fatty acids; SFA, saturated fatty acids.

^1^ *n* = 3–5 beetles per treatment at each time point.

^2^Means not followed by the same superscripted letter are significantly different (*p* < .05).

^3^ Calculated as (20:4n‐6/[20:4n‐6 + 18:2n‐6]).

^4^ Calculated as (16:1c9 + 18:1c9/[16:1c9 + 18:1c9 + 16:0 + 18:0]).

The analysis of whole‐body fatty acids among different collection dates within adult *L. decemlineata* obtained from commercially managed potato illustrate several differences over time (Table 3). The main fatty acid classes, total MUFA, and PUFA were significantly decreased and increased, respectively, in later‐emerging *L. decemlineata* (*p* = .01 and .03, respectively). The most consistent and predominant change among the MUFAs was oleic acid (OLA, 18:1c9), with a decrease of 23.9% from Week 1 to Week 5 of emergence. Total PUFA increased 16.6% from Week 1 to Week 5, with linoleic acid (LNA; 18:2n‐6), α‐linolenic acid (18:3n‐3), and ARA (20:4n‐6) all increasing over time. The Δ^9^ index decreased over time (*p* = .07), while the Δ^6^‐desaturase index significantly increased over time (*p* < .01). The pool of unidentified compounds also significantly increased over time (*p* < .01). The most abundant unknown compound existed at 2.5% of the total peak areas, while the remaining compounds representing the total unknowns were distributed in low abundance (<0.5% of total peak area).

**Table 3:** Commercial *L. decemlineata* population fatty acid composition over time

Week	1	2	3	4	5	*p* value
Fatty acid	Relative abundance (g/100 g FAME)					
14:0	1.3^b^	1.6^b^	2.4^a^	1.6^ab^	5.3^ab^	.04
16:0	13.6^a^	11.5^ab^	11.7^ab^	9.8^b^	6.4^c^	.01
18:0	5.2	6.2	6.4	8.7	8.2	.20
20:0	0.7^b^	1.0^ab^	1.0^ab^	1.5^a^	1.5^a^	**<**.01
∑SFA	20.8	20.4	21.7	21.7	21.5	.27
16:1 c9	0.7	0.6	0.7	0.4	0.5	.73
18:1 c9	47.3^a^	40.4^ab^	36.8^ab^	32.1^b^	22.4^c^	.01
18:1 c11	0.3^c^	1.9^a^	1.2^b^	1.1^abc^	1.6^ab^	.05
∑MUFA	48.3^a^	41.8^ab^	38.4^ab^	33.6^b^	24.4^c^	.01
18:2n‐6	13.4	15.2	17.5	19.0	22.8	.19
18:3n‐3	11.4^c^	13.0^c^	13.7^bc^	15.6^a^	15.7^ab^	.02
20:4n‐6	0.6^b^	0.7^b^	0.8^b^	1.2^ab^	2.0^a^	.03
∑PUFA	25.7^c^	28.9^bc^	33.1^abc^	36.9^ab^	42.3^a^	.03
Unknown	5.2^b^	8.7^ab^	6.8^b^	7.6^b^	11.9^a^	**<**.01
Δ^6^ index^3^	0.04^b^	0.03^b^	0.04^b^	0.06^b^	0.08^a^	**<**.01
Δ^9^ index^4^	0.71	0.69	0.67	0.63	0.61	.07

Abbreviations: FAME, fatty acid methyl esters; MUFA, monounsaturated fatty acids; PUFA, polyunsaturated fatty acids; SFA, saturated fatty acids.

^1^ *n* = 3–5 beetles per treatment at each time point.

^2^Means not followed by the same superscripted letter are significantly different (*p* < .05).

^3^ Calculated as (20:4n‐6/[20:4n‐6 + 18:2n‐6]).

^4^ Calculated as (16:1c9 + 18:1c9/[16:1c9 + 18:1c9 + 16:0 + 18:0]).

### 3.3 Differential transcript abundance analysis

**Figure 1:**
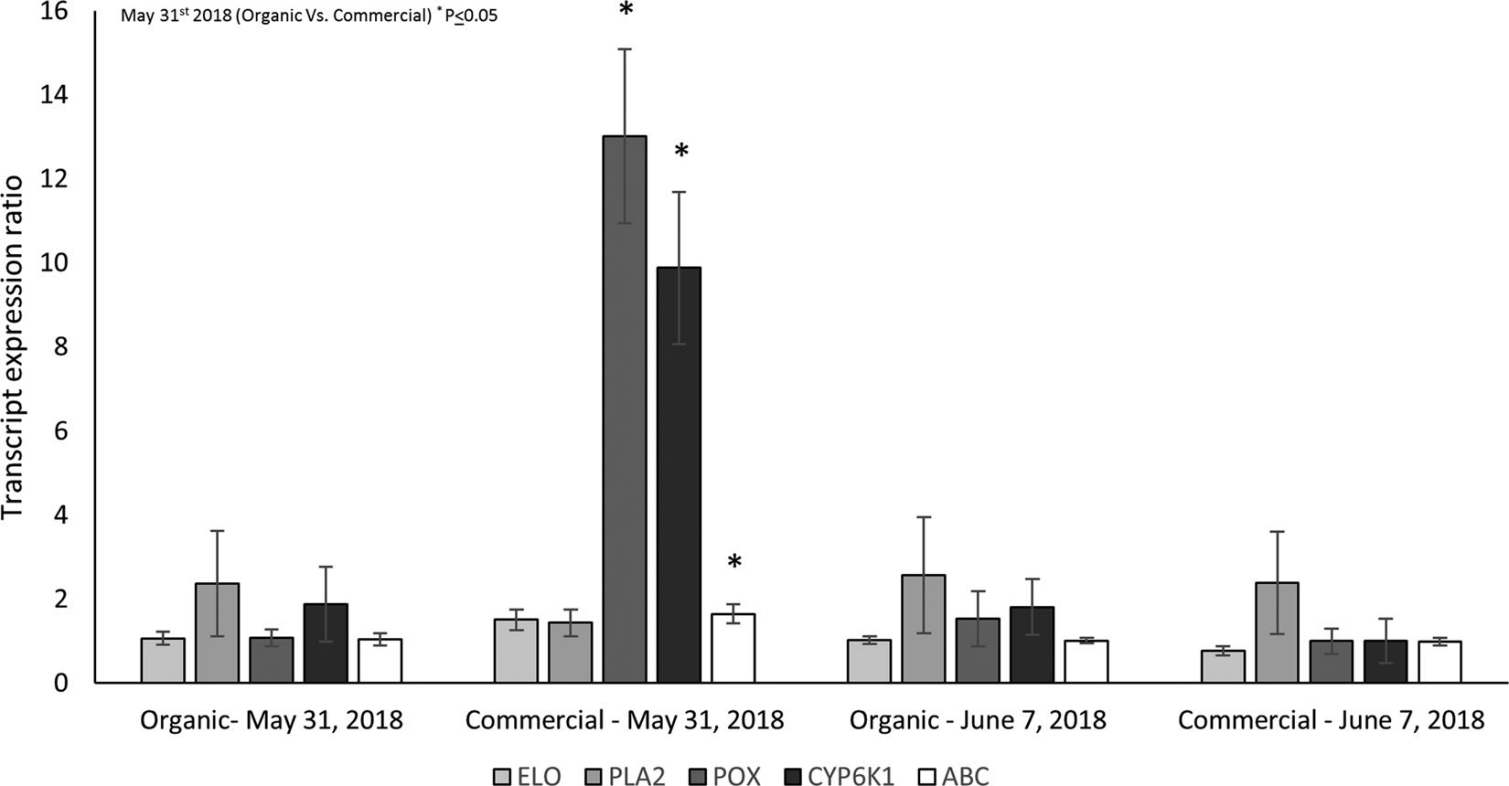
Transcript expression of ATP‐binding cassette subfamily G (ABC), cytochrome P450 6k1 (CYP6K1), elongation of very long fatty acids (ELO), phospholipase A2‐like (PLA2), and peroxidase‐like (POX) of organic and commercial *Leptinotarsa decemlineata*. ATP, adenosine triphosphate

Transcript expression of cytochrome P450 6K1, peroxidase‐like, and an ATP‐binding cassette subgroup family G were all overexpressed in the commercially managed, insecticide‐resistant population of *L. decemlineata*. Transcript expression of five unique targets was compared between the insecticide susceptible and resistant populations. The targets compared include an ATP‐binding cassette subfamily G, cytochrome p450 6k1, elongation of very long fatty acids, phospholipase A2‐like, and peroxidase‐like. Expression was observed at two unique time points during the emergence phenology of insects (May 31, 2018 and Jun 7, 2018; Week 1 and Week 2 postemergence). Transcript expression ratios of targets can be seen in Figure 1. There was a significant difference in transcript abundance for the cytochrome P450 6k1, the peroxidase gene, and the ATP‐binding cassette between the commercially managed, insecticide‐resistant population and the organically managed, suseptible population at the first time point.

### 3.4 *L. decemlineata* emergence phenology

**Figure 2:**
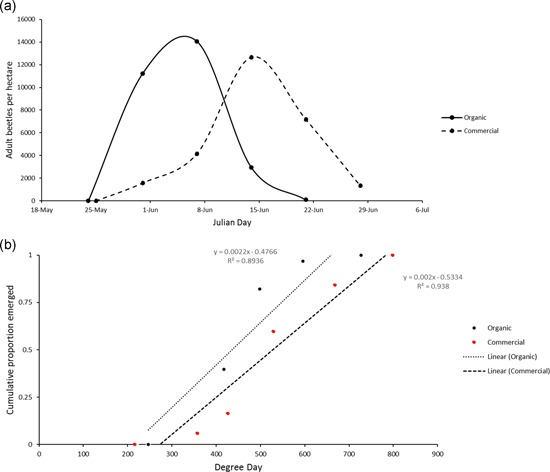
(a) Count per hectare of diapause emerging *L. decemlineata* from two distinct populations of *L. decemlineata* (organic and commercial. (b) Cumulative proportion of beetle emergence plotted over degree days

Temporal patterns of adult *L. decemlineata* emergence in the commercially managed potato fields were notably prolonged in comparison to the organically managed field population. Both field populations obtained in Wisconsin had similar planting dates and similar degree day accumulations (base 52°F; Figure 2 ). The organically managed population, with no prior history of systemic insecticide use, showed an earlier peak in emergence time, while the population with a history of systemic insecticide inputs had a more protracted period of emergence, with a delayed peak in emergence timing (Figure 2 a). When emergence phenology was plotted in terms of cumulative proportion emerged over degree days accumulated, there was a notable shift in the time required for 50% of adults to emerge within the commercially managed, insecticide‐resistant population (Figure 2 b). Specific estimates for the median emergence time in degree days for the susceptible population to emerge was 444 DD_52_ and the resistant population was 517 DD_52_.

## 4 DISCUSSION

*L. decemlineata* is an agricultural pest of significant concern in many potatoes producing regions throughout the world due to their high fecundity rates, defoliation capacity in solanaceous crops, and their ability to develop resistance to insecticides used for their control. Beetle populations have developed insecticide resistance to all major classes of insecticides registered for their control (Whalon et al., 2019). Multiple physiological and biological processes (enzymatic detoxification, cross‐resistance, cuticular hardening, mutation in reception sites, and behavioral adaptations) have been proposed to be partially responsible for increased insecticide resistance, and it has been hypothesized that *L. decemlineata* has been selected to resist insecticides through deferentially expressing multiple mechanisms in different geographic locations (Alyohkin et al., 2008). We examined two populations of *L. decemlineata* in Wisconsin that have experienced vastly different insecticide regimens. The populations corresponded to an organically managed potato field using only USDA National Organic Program and Organic Management Review Institute‐approved standards and inputs for *L. decemlineata* control, (which excluded the use of any systemic insecticides, including neonicotinoids), together with a commercially managed potato field that has used at‐plant, systemic neonicotinoid insecticides for more than 20 years. The commercially managed population in Wisconsin has been previously determined to be resistant to neonicotinoid insecticides (Clements et al., 2016b; Crossley, Rondon, & Schoville, 2018; Huseth & Groves, 2013). Half lethal concentration assays were conducted to observe *L. decemlineata* susceptibility to imidacloprid throughout their emergence phenology. Insects from the organically managed field had LC_50_ values that ranged from 1.64 to 2.82 µg of imidacloprid throughout the first‐generation emergence phenology, while insects from the commercial population ranged from 2.00 to 12.12 µg of imidacloprid, confirming that the commercially managed population has a more insecticide‐resistant phenotype in response to imidacloprid.

The fatty acid composition analyses suggest specific associations between fatty acid metabolism and imidacloprid‐resistance could be correlated, and that the role of fatty acids in increasing *L. decemlineata* insecticide resistance deserves further investigation. More specifically, the increased Δ^6^‐desaturase index coinciding with a decreased Δ^9^‐desaturase index over time in the commercially managed population is interesting from a mechanistic standpoint. These data suggest imidacloprid resistant *L. decemlineata* may be sacrificing energy storage in favor of upregulating arachidonic acid and potentially eicosanoid‐related metabolism. OLA, a primary product of Δ^9^‐desaturase, typically represents the most abundant fatty acid in the fat‐body tissue of *L. decemlineata* (and other Coleopterans) and is typically synthesized de novo or obtained directly from the diet (Arrese, Gazard, Flowers, Soulages, & Wells, 2001; Lehmann et al., 2012; Ogg, Meinke, Howard, & Stanley‐Samuelson, 1993). Whole‐body OLA is typically reported as being the most abundant fatty acid in prediapausing Coleopterans and is significantly reduced through—and following—the diapause period as energy is spent before feeding (Lambremont, Blum, & Schrader, 1964). Reports on the changes in *L. decemlineata* fatty acids that occur postemergence suggest OLA increases over time as beetles consume foliage and build fat‐body stores (Arrese et al., 2001; Lehmann et al., 2012). In agreement with previous reports, OLA in imidacloprid susceptible *L. decemlineata* did not significantly change over time. However, OLA in the insecticide‐resistant, commercially managed population of *L. decemlineata* decreased by more than half over increasing emergence times. The decrease in the Δ^9^‐desaturase index over time in the resistant population suggests that this population may be decreasing de novo synthesis of energy‐storing fatty acids (Sampath & Ntambi, 2006). Together, the data suggest a possible mechanism whereby resistant *L. decemlineata* may be sacrificing energy storage for other purposes, perhaps toward upregulation of insecticide detoxification systems or a general increase in fatty acid β‐oxidation for increased energy expenditure for metabolic processes. The associated increase in total PUFA, LNA, and ARA with decreased OLA provides evidence that OLA depletion may be coordinated with an increase in the metabolism of LNA to ARA and potentially the upregulation of oxylipin production. Though direct evidence of oxylipin (e.g., eicosanoid) production was not obtained, the increase in Δ^6^‐desaturase rate over time in resistant *L. decemlineata* suggests an increased conversion of LNA to ARA in resistant beetles. Eicosanoid production is, therefore, implicated in insecticide resistance, as increasing ARA provides a more abundant pool of substrate for enzymes involved in eicosanoid formation, namely lipoxygenases, cyclooxygenases, and cytochrome P450s (recently reviewed by Hanna & Hafez, 2018). While many of these enzymes have been implicated previously in insecticide resistance, little is known on the regulation and precise involvement of these enzymes in *L. decemlineata* resistance (Büyükgüzel, Hyršl, & Büyükgüzel, 2010; Yamamoto et al., 2013b; Yamamoto, Ichinose, Aso, Udono, & Katakura, 2013a).

We investigated the gene expression of a very‐long‐chain fatty acid elongase, phospholipase A2, CYP6K1, and peroxidase, which are all involved in the ARA‐eicosanoid metabolic cascade. Our results demonstrate that both CYP6K1 and peroxidase are correlated with insecticide resistance status. Early expression at Week 1 in the resistant group (commercially managed population) may be an indicator that later‐emerging groups have an upregulated abundance of these enzymes to aid in insecticide resistance. It is possible that isoforms other than those analyzed are modulated, as animal species have various isoforms of these enzymes utilizing different lipid classes and fatty acid chain lengths as substrates. Further, characterization of eicosanoid production pathways in *L. decemlineata* would improve our understanding of these processes and their putative role in xenobiotic metabolism. In addition, CYP6K1 gene expression was upregulated in the commercially managed population at the first collection time point when in‐plant, systemic concentrations of insecticide would be at its highest, however, it was not overexpressed in the second time point examined. This could suggest that this CYP6k1 is being induced, but not constitutively expressed throughout the population, and may regulate fatty acid metabolism. Further, it has been established that *L. decemlineata* possess multiple modes of insecticide resistance, and while we have only examined metabolic detoxification and behavioral resistance in the current investigation, it is plausible that *L. decemlineata* may also be using other forms of insecticide resistance to combat the insecticide insults.

Acknowledging the limitations of this study, the observed differences in fatty acid composition over time may be due, in part, to contribution from factors unrelated to insecticide resistance. First, beetle weights and fat content were not assessed in this study. It is, therefore, unclear whether resistant *L. decemlineata* had decreased total fat‐body lipid at later emergence times compared to the susceptible (organically managed) population. Understanding lipid content in both beetle populations would further clarify whether *L. decemlineata* is beta‐oxidizing fat stores or modulating their fatty acid composition within fixed body fat pools. Second, the foliage fatty acid compositions contributing to insect diets from each population site are unknown. Differences in *L. decemlineata* composition between resistant and susceptible groups may be affected by dietary fatty acid differences. Because of this, we did not compare site‐specific fatty acid differences between *L. decemlineata* populations, but rather investigated change over time within each population. Knowledge of foliar fatty acid compositions would help to clarify whether the increased levels of LNA and ARA in resistant beetles are due to increased LNA intake from foliage or the direct result of *L. decemlineata* fatty acid metabolism. The increased Δ^6^‐desaturase index, however, is independent of fatty acid intake since ARA is not obtained through potato foliage.

The current investigation further revealed a difference in temporal patterns of *L. decemlineata* emergence between the two populations of *L. decemlineata* examined. The population of insects deemed as insecticide‐resistant emerged later in the growing season and over a longer period of time. This shift is demonstrated with the change in the number of degree days required for the emergence of half of the cumulative population per degree day, as the resistant population emerged 72‐degree days later than the susceptible population. Further, investigations are needed to confirm that these trends are accurately correlated to a resistant phenotype, and not just an artifact of the specific fields examined in the current study. These data are suggestive of later emergence in the resistant population, resulting in the emergence of a lower insecticide concentration in potato foliage (Huseth et al., 2014).

In the current study, we observed a delayed emergence time in an insecticide‐resistant population of *L. decemlineata* compared to an insecticide susceptible population. The behavioral adaptation most likely has arisen as a response to systemic applications of neonicotinoid insecticides applied at potato planting. The delayed emergence phenology was associated with evidence of decreased beetle lipid storage, increased ARA synthesis, and increased CYP, POX, and ABC gene expression, suggesting a role for directed fatty acid metabolism in promoting insecticide resistance. As insecticide resistance continues to be a major concern for pest managers, the agricultural community will need to consider all forms of insecticide resistance, including the ability to evolve variable emergence phenology to avoid insecticide exposure and the underlying mechanisms that regulate detoxification of insecticides.

## Supporting information

Supporting information
