# Supplementary material for: Changes in emergence phenology, fatty acid composition, and xenobiotic‐metabolizing enzyme expression is associated with increased insecticide resistance in the Colorado potato beetle
Source: Arch Insect Biochem Physiol. 2019 Oct 16;103(3):e21630. doi: 10.1002/arch.21630 (PMC7027459; doi:10.1002/arch.21630)
Supplement: Supplementary file 1 — Supporting information [file ARCH-103-e21630-s001.docx]

# Supplementary Information

**Supplementary Table S1:** Quantitative PCR primers and primer efficiencies.

|  | **Forward Primer (5’-3’)** | **Reverse Primer (5’-3’)** | **Primer Efficiency** | **Targeted Gene** |
| --- | --- | --- | --- | --- |
| RP4 | AAAGAAACGAGCATTGCCCTTCCG | TTGTCGCTGACACTGTAGGGTTGA | 1.93 | Ribosomal protein 4 |
| ABC | GGGAGCAACCGGTTCAATCT | CCCAATTTCAAGTTGGCAGCA | 1.96 | ATP-binding cassette subfamily G |
| CYP | TGCTGAAAGACCTGGAAGTG | CTCATCATGGGAAGAAGACTGG | 2.01 | Cytochrome P450 6k1 |
| ELO | GCCTTCAAATCCACTGCTTTAC | CCGAGTTTCCACACAAACATATC | 1.95 | Elongation of very long fatty acids |
| PLA2 | TCGGCTTAGATGCAAGATCAG | GCAATATTTCCAGCACCACAC | 2.12 | Phospholipase A2-like |
| POX | CCTGTCCGACCACCAAATAC | TTGCAGGAACCAGCCTTAC | 1.93 | Peroxidase-like |

**Supplementary Table S2:** Whole-body fatty acid composition and SEMs of *L. decemlineata* populations within each week

|  | Susceptible | | | | Resistant | | | |  |
| --- | --- | --- | --- | --- | --- | --- | --- | --- | --- |
| Week | 1 | 2 | 3 | 4 | 1 | 2 | 3 | 4 | 5 |
| Fatty acid | *Relative abundance (g/100g FAME)* | | | | | | | | |
| 14:0 | 1.3  (0.2) | 2.2  (0.2) | 2.4  (0.2) | 0.6  (0.2) | 1.3  (0.1) | 1.6  (0.1) | 2.4  (0.3) | 1.6  (0.2) | 5.3  (2.1) |
| 16:0 | 11.2  (0.8) | 11.6  (1.1) | 9.3  (1.1) | 12.4  (1.7) | 13.6  (1.0) | 11.5  (1.5) | 11.7  (1.3) | 9.8  (0.80) | 6.4  (1.1) |
| 18:0 | 7.7  (1.0) | 5.8  (0.7) | 7.9  (1.2) | 6.1  (1.0) | 5.2  (0.4) | 6.2  (1.1) | 6.4  (1.1) | 8.7  (1.6) | 8.2  (1.5) |
| 20:0 | 1.3  (0.2) | 1.3  (0.3) | 1.4  (0.1) | 1.2  (0.2) | 0.7  (0.06) | 1.0  (0.23) | 1.0  (0.15) | 1.5  (0.20) | 1.5  (0.31) |
| ∑SFA | 21.5  (0.3) | 21.0  (0.3) | 21.0  (0.4) | 20.3  (1.1) | 20.8  (0.8) | 20.4  (0.4) | 21.7  (0.7) | 21.7  (1.4) | 21.5  (0.3) |
|  |  |  |  |  |  |  |  |  |  |
| 16:1 c9 | 0.4  (0.12) | 0.8  (0.12) | 0.4  (0.16) | 0.8  (0.16) | 0.7  (0.19) | 0.6  (0.17) | 0.7  (0.20) | 0.4  (0.08) | 0.5  (0.10) |
| 18:1 c9 | 37.0  (4.4) | 32.9  (2.3) | 29.0  (2.1) | 38.6  (5.8) | 47.3  (4.6) | 40.4  (4.3) | 36.8  (4.4) | 32.1  (3.1) | 22.4  (2.1) |
| 18:1 c11 | 1.6  (0.2) | 1.3  (0.1) | 1.8  (0.2) | 1.2  (0.4) | 0.3  (0.2) | 1.9  (0.2) | 1.2  (0.2) | 1.1  (0.5) | 1.6  (0.5) |
| ∑MUFA | 38.7  (4.1) | 35.0  (2.3) | 31.2  (2.3) | 40.3  (5.6) | 48.3  (4.5) | 41.8  (4.0) | 38.4  (4.3) | 33.6  (2.9) | 24.4  (2.4) |
|  |  |  |  |  |  |  |  |  |  |
| 18:2 n-6 | 17.3  (2.0) | 21.6  (1.1) | 22.4  (2.3) | 18.2  (4.2) | 13.4  (2.9) | 15.2  (1.5) | 17.5  (2.3) | 19.0  (2.9) | 22.8  (3.7) |
| 18:3 n-3 | 11.5  (0.7) | 12.2  (0.3) | 13.2  (0.7) | 12.7  (1.7) | 11.4  (1.3) | 13.0  (0.6) | 13.7  (0.4) | 15.6  (0.8) | 15.7  (0.9) |
| 20:4 n-6 | 0.6  (0.1) | 1.1  (0.2) | 1.0  (0.1) | 0.9  (0.3) | 0.6  (0.2) | 0.7  (0.3) | 0.8  (0.2) | 1.2  (0.4) | 2.0  (0.3) |
| ∑PUFA | 32.0  (2.8) | 35.9  (1.5) | 37.5  (2.1) | 33.0  (5.2) | 25.7  (4.0) | 28.9  (2.3) | 33.1  (3.0) | 36.0  (3.3) | 42.3  (3.6) |
|  |  |  |  |  |  |  |  |  |  |
| Unknown | 9.9  (1.8) | 8.4  (1.1) | 10.4  (1.5) | 6.5  (1.1) | 5.2 (1.3) | 8.7  (1.8) | 6.8  (1.5) | 7.6  (1.2) | 11.9  (1.09) |
| Δ^6^ index^3^ | 0.03  (0.002) | 0.05  (0.006) | 0.04  (0.004) | 0.05  (0.007) | 0.04 (0.005) | 0.03 (0.004) | 0.04 (0.009) | 0.06 (0.011) | 0.08 (0.005) |
| Δ^9^ index^4^ | 0.66  (0.03) | 0.66  (0.01) | 0.63  (0.02) | 0.67  (0.03) | 0.71 (0.02) | 0.69 (0.02) | 0.67 (0.03) | 0.63 (0.03) | 0.61 (0.02) |

^1^ n=3-5 beetles per treatment at each time point.

^2^ Values represent group means (±SEM).

^3^ Calculated as [20:4n-6 / (20:4n-6 + 18:2n-6)].

^4^ Calculated as [16:1c9 + 18:1c9 / (16:1c9+ 18:1c9 + 16:0 + 18:0)].
